# Automated machine learning for predicting perioperative ischemia stroke in endovascularly treated ruptured intracranial aneurysm patients

**DOI:** 10.3389/fneur.2025.1599856

**Published:** 2025-06-19

**Authors:** Yuhang Peng, Ke Bi, Xiaolin Zhang, Ning Huang, Xiang Ji, Weifu Chen, Ying Ma, Yuan Cheng, Yongxiang Jiang, Jianhe Yue

**Affiliations:** ^1^Department of Neurosurgery, The Second Affiliated Hospital of Chongqing Medical University, Chongqing, China; ^2^Department of Emergency, The Second Affiliated Hospital of Chongqing Medical University, Chongqing, China

**Keywords:** complication prediction, automated machine learning, endovascular therapy, intracranial aneurysms, perioperative ischemia stroke

## Abstract

**Objective:**

This study aims to develop and validate an automated machine learning model to predict perioperative ischemic stroke (PIS) risk in endovascularly treated patients with ruptured intracranial aneurysms (RIAs), with the goal of establishing a clinical decision-support tool.

**Methods:**

In this retrospective cohort study, we analyzed RIA patients undergoing endovascular treatment at our neurosurgical center (December 2013–February 2024). The least absolute shrinkage and selection operator (LASSO) method was used to screen essential features associated with PIS. Based on these features, nine machine learning models were constructed using a training set (75% of participants) and assessed on a test set (25% of participants). Through comparative analysis, using metrics such as area under the receiver operating characteristic curve (ROCAUC) and Brier score, we identified the optimal model—random forest (RF)—for predicting PIS. To interpret the RF models, we utilized the Shapley Additive exPlanations (SHAP).

**Results:**

The final cohort comprised 647 consecutive RIA patients who underwent endovascular intervention. LASSO regression identified 13 clinically actionable predictors of PIS from the initial variables. These predictors encompassed: vascular risk factors (hyperlipidemia, arteriosclerosis); neuroimaging indicators of severity (modified Fisher scale, aneurysm location, and neck-to-diameter ratio); clinical status (Glasgow Coma Scale score, Hunt-Hess grade, age, sex); procedural complications (intraprocedural rupture, periprocedural re-rupture); and therapeutic determinants (therapy method and history of ischemic comorbidities). Nine machine learning algorithms were evaluated using stratified 10-fold cross-validation. Among them, the RF model demonstrated the best performance, with the ROCAUC of 92.11% (95%CI: 89.74–94.48%) on the test set and 87.08% (95%CI: 81.23–92.93%) on the training set. Finally, in a prospective validation cohort, the RF predictive model demonstrated an accuracy of 88.23% in forecasting the incidence of PIS. Additionally, based on this predictive model, this study developed a highly convenient web-based calculator. Clinicians only need to input the patient’s key factors into this calculator to predict the postoperative incidence of PIS and provide individualized treatment plans for the patient.

**Conclusion:**

We successfully developed and validated an interpretable machine learning framework, integrated with a clinical decision-support system, for predicting postprocedural PIS in endovascularly treated RIAs patients. This tool effectively predicted the likelihood of PIS, enabling high-risk patients to promptly take specific preventive and therapeutic measures.

## 1 Introduction

Intracranial aneurysms (IAs) are abnormal dilations of blood vessels within the brain, characterized by degenerative changes across all layers of the vessel wall, leading to vasodilation (1). In adults over the age of 50, the prevalence of IAs is estimated to be approximately 2% (2). The onset of IAs is typically insidious, as they rarely present with obvious symptoms or clinical signs prior to rupture. The most critical risk posed by IAs is rupture, which can result in catastrophic hemorrhage. Ruptured intracranial aneurysms (RIAs) constitute a severe neurosurgical emergency, with an annual incidence of 6–9 cases per 100,000 individuals. The associated mortality rate approaches 35%, while a significant proportion of survivors sustain permanent neurological deficits, leading to long-term disability (3). Currently, treatment options for aneurysms primarily consist of surgical clipping and endovascular treatment. Endovascular treatment has emerged as the preferred choice due to its minimally invasive nature and quick recovery time. Consequently, its role in the treatment of RIAs has become increasingly prominent in recent years (4, 5).

Despite the advantages of endovascular treatment—namely its minimally invasive nature and rapid recovery—the occurrence of perioperative complications cannot be ignored. Each year, 310 million people worldwide undergo surgery, with more than 600,000 experiencing perioperative strokes. The majority of studies focus on ischemic stroke, which accounts for approximately 95% of perioperative strokes, compared to approximately 5% for hemorrhagic stroke (6, 7). Studies demonstrate that perioperative ischemic stroke (PIS) is the primary cause of disability and death following RIA treatment (8–10). The expert consensus of the American Society of Neurosurgical Anesthesiology and Critical Care Sciences defines PIS as an ischemic cerebral infarction that occurs during or within 30 days after the operation (11). With the growing number of patients undergoing endovascular surgery, the occurrence of PIS has attracted increasing attention (12–14). Relevant studies have shown that multiple factors (such as hypertension, previous stroke, diabetes, and aneurysm morphology) can increase the risk of PIS. However, determining which of these risk factors require clinical intervention or prevention strategies remains a major challenge (15–17). Recent studies suggest that integrating machine learning techniques into predictive models can substantially improve the accuracy of risk stratification (18–20). Therefore, a systematic synthesis of risk factors associated with PIS following endovascular treatment is essential. By developing robust machine learning-based predictive models, we can enhance the application scope and practicality of risk prediction models in clinical practice. This will enable clinicians to make decisions with more sufficient data support, thereby reducing the incidence of PIS in RIA patients after treatment and achieving the goal of improving long-term prognosis.

In this study, nine distinct prediction models for PIS were developed using machine learning methods, leveraging data from 647 patients who underwent procedures. Through comparative analysis, the most effective predictive model for identifying high-risk patients was identified, facilitating its clinical application. To assist healthcare professionals in effectively utilizing this model, we have also developed a web-based calculator using the collected data. This tool enables the identification of high-risk patients and provides them with more effective treatment options.

## 2 Methods

### 2.1 Source and extraction of data

The design and execution of this retrospective investigation followed the STROBE Guidelines (21, 22). Two investigators independently collected data from patients with RIAs who underwent endovascular treatment at the Second Affiliated Hospital of Chongqing Medical University from 25 December 2013 to 1 February 2024. The data included basic information (age, gender, drinking history, smoking history, hypertension, coronary heart disease, etc.), aneurysm information (size, location, etc.), and treatment method. Approval for this study was obtained from the Ethics Committee of our hospital, and all patient data were anonymized. No patient-specific information was retained, and consent was obtained from the patients.

To further verify the accuracy of the models, prospective data were collected at the Second Affiliated Hospital of Chongqing Medical University from 12 February 2024 to 1 May 2024, focusing on RIA patients who underwent endovascular treatment data.

Inclusion criteria:

Patients were included in the study if they met the following conditions:

1. The aneurysms were saccular;
2. Patients with aneurysms treated with endovascular coiling (EC), stent-assisted coiling (SAC), or flow-diverted (FD) stents;
3. Patients aged 18–85 years, not pregnant, and fully competent to act.

Exclusion criteria:

Patients were excluded from the study if they met any of the following:

1. The aneurysms were non-saccular (e.g., dissecting, fusiform, traumatic, and blood blister-like aneurysms);
2. Patients treated with hybrid clipping and endovascular procedures;
3. Patients who were adolescents, aged 85 years or older, pregnant, lacking voluntary capacity, or had limited decision-making capacity.

### 2.2 Endovascular procedures

The neurovascular team decided on specific endovascular therapy plans tailored to each patient. Procedures involved endovascular surgery following general anesthesia. To avoid embolic occurrences, systemic intravenous heparin systemic intravenous heparin was administered during the procedure. For patients with RIAs, oral antiplatelet agents were not administered after surgery if EC was selected. If SAC was chosen, patients received 300 mg of aspirin and 300 mg of clopidogrel after surgery. Starting the next day, the regimen was changed to aspirin 100 mg/d and clopidogrel 75 mg/d. The patient was advised to take clopidogrel 75 mg/d for 3 weeks and aspirin 100 mg/d for 6 months. If FD treatment was selected, patients were given 300 mg of aspirin and 300 mg of clopidogrel postoperatively, and the next day, clopidogrel 75 mg/d for 6 months and long-term aspirin 100 mg/d.

### 2.3 Outcome measures

We define PIS as any adverse neurological events that occur during or within 30 days after surgery in RIA patients receiving endovascular treatment. Computerized tomography (CT) examinations were conducted directly after the operation, and repeated examinations were performed for cases with deteriorated neurological function. When CT images do not confirm suspected new-onset cerebral infarction, magnetic resonance imaging (MRI) examination is performed (16, 23). Patients confirmed by both clinical symptoms and imaging were included in the complication group, and those without were included in the control group.

### 2.4 Model input features

We considered 24 potential factors, encompassing age, gender, alcohol and tobacco use history, hypertension, arteriosclerosis, hyperlipidemia, diabetes, previous cerebral ischemia, Glasgow Coma Scale (GCS), modified Fisher scale, Hunt and Hess grade, oculomotor nerve palsy, aneurysm location, neck, length, maximum diameter, ratio of aneurysm neck to maximum diameter, multiple aneurysms, perioperative re-rupture, preoperative intracranial hematoma, intraprocedural rupture (IPR), time from symptom onset to surgery, treatment method (EC, SAC, or FD), and the occurrence of PIS. Subsequently, we used the least absolute shrinkage and selection operator (LASSO) to identify factors associated with PIS (24).

### 2.5 Statistical analysis

The missing data were treated in the following ways: groups were eliminated if the percentage of missing values was less than 5%, random forest (RF) regression was used for interpolation if the percentage of missing values was between 5 and 20%, and if the percentage of missing values was greater than 20%, the group was removed from the final complete dataset. It can be seen from Supplementary Figure 1 that there are no missing values in our data, so no special processing has been carried out. R software, version 4.3.2, was used for all analyses. The distribution of continuous variables, which were reported as mean ± standard deviation (SD), was tested for normality using the Shapiro–Wilk test; the independent sample *t*-test was used for comparison. The continuous variables with a skewed distribution were represented by the quartile and median (IQR). Frequencies and percentages were utilized to represent the categorical variables, and Fisher’s exact probability method or the chi-squared test was applied for statistical analysis. Nine models were created based on the feature set of the most significant characteristics after the most significant features were filtered out using LASSO regression analysis.

Nine learning models—Decision Tree (DT), Light Gradient Boosting Machine (LightGBM), eXtreme Gradient Boosting (XGBOOST) algorithm, Logistic Regression (LR), RF, Elastic Net (ENet), Multi-layer Perceptron (MLP), Support Vector Machine (SVM), and K-Nearest Neighbor machine (KNN)—were constructed in order to achieve the best predictive performance. Area under the receiver operating characteristic curve (ROCAUC) and Brier scores were used to assess the model’s performance. Clinical decision curve analysis (DCA) evaluation model’s clinical viability (25). The relationship between each attribute and PIS was shown using R’s Shapley Additive package (SHAP) tool once the optimal model had been obtained. The best model will finally be built on a web-based calculator using the shiny package (26, 27).

## 3 Results

### 3.1 Patient characteristics

This study enrolled 647 patients with RIAs treated with endovascular therapy (details shown in Table 1), comprising 424 females (65.5%) and 223 males (34.5%) with a median age of 54.9 years. Treatment modalities included EC (*n* = 199, 30.8%), SAC (*n* = 381, 58.9%), and FD stents (*n* = 67, 10.3%). Aneurysms were predominantly located in the anterior communicating artery (AcomA, 27.5%) and the posterior communicating artery (PcomA, 27.5%), followed by the middle cerebral artery (MCA, 15.8%), internal carotid artery (ICA, 11.1%), anterior cerebral artery (ACA, 4.9%), vertebral artery (VA, 3.2%), posterior inferior cerebellar artery (PICA, 2.3%), posterior cerebral artery (PCA, 2.3%), choroidal artery (ChoA, 2.6%), basilar artery (BA, 1.4%), and ophthalmic artery (OA, 1.2%).

**Table 1:** Comparison of variables between the complication group and the control group.

Variables	All (*n* = 647)	Complication group (*n* = 166)	Control group (*n* = 481)	*p*-Value
Age, median (Q1, Q3)	54.9 (19, 53)	59.3 (39, 59)	53.5 (19, 52)	<0.001
Sex, n%				0.002
Male	223 (34.467)	42 (25.301)	181 (37.630)	
Female	424 (65.533)	124 (74.699)	300 (62.370)	
Previous ischemic comorbidity, n%				<0.001
Have	45 (6.955)	23 (13.855)	22 (4.574)	
Not	602 (93.045)	143 (86.145)	459 (95.426)	
Arteriosclerosis, n%				<0.001
Have	234 (36.167)	81 (48.795)	153 (31.809)	
Not	413 (63.833)	85 (51.205)	328 (68.191)	
Hypertension, n%				<0.001
Have	262 (40.495)	82 (49.396)	180 (37.422)	
Not	385 (59.505)	84 (50.624)	301 (62.578)	
Hyperlipidemia, n%				<0.001
Have	441 (68.161)	147 (88.554)	294 (61.123)	
Not	206 (31.839)	19 (11.445)	187 (38.877)	
Diabetes, n%				0.744
Have	40 (6.182)	11 (6.627)	29 (6.029)	
Not	607 (93.818)	155 (93.373)	452 (93.971)	
Smoking, n%	156 (24.111)	38 (22.892)	118 (24.532)	<0.001
Alcohol, n%	67 (10.355)	17 (10.241)	50 (10.395)	<0.001
Oculomotor nerve palsy, n%				0.036
Have	37 (5.719)	8 (4.819)	29 (6.029)	
Not	610 (94.281)	158 (95.181)	452 (93.971)	
Hunt and Hess Grade, n%				<0.001
I	279 (43.122)	28 (16.867)	251 (52.183)	
II	280 (43.277)	83 (50.000)	197 (40.596)	
III	63 (9.737)	33 (19.880)	30 (6.237)	
IV	25 (3.864)	22 (13.253)	3 (0.624)	
Modified Fisher scale, n%				<0.001
I	276 (42.658)	22 (13.253)	254 (52.807)	
II	190 (29.366)	45 (27.108)	145 (30.146)	
III	100 (15.456)	45 (27.108)	51 (10.603)	
IV	81 (12.519)	50 (30.120)	31 (6.445)	
GCS, median (Q1, Q3)	13.9 (3, 15)	12.7 (3, 14)	14.3 (5, 15)	<0.001
Perioperative aneurysm re-rupture, n%				<0.001
Have	58 (8.964)	38 (22.892)	20 (4.158)	
Not	589 (91.036)	128 (77.108)	461 (95.842)	
Aneurysm location, n%^*^				<0.001
AcomA	178 (27.512)	37 (22.289)	141 (29.314)	
PcomA	178 (27.512)	43 (25.904)	135 (28.067)	
ACA	32 (4.946)	10 (6.024)	22 (4.574)	
MCA	102 (15.765)	29 (17.470)	73 (15.177)	
PCA	15 (2.318)	2 (1.205)	13 (2.703)	
ICA	72 (11.128)	17 (10.241)	55 (11.435)	
ChoA	17 (2.628)	8 (4.819)	9 (1.871)	
PICA	15 (2.318)	3 (1.807)	12 (2.495)	
VA	21 (3.246)	12 (7.229)	9 (1.871)	
BA	9 (1.391)	4 (2.410)	5 (1.040)	
OA	8 (1.237)	1 (0.604)	7 (1.455)	
Aneurysm neck, median (Q1, Q3)	4.15 (0.6, 3.6)	4.41 (1, 3.5)	4.07 (0.6, 3.6)	<0.001
Aneurysm maximum diameter, median (Q1, Q3)	4.82 (0.7, 4.2)	4.85 (2.7, 5.6)	3.6 (0.7, 4.2)	<0.001
Ratio of aneurysm neck to maximum	0.945 (0.12, 0.84)	1.05 (0.32, 0.88)	0.911 (0.12, 0.83)	<0.001
Diameter, median (Q1, Q3)				
Multiple aneurysms, n%				0.039
Have	157 (24.266)	51 (30.723)	106 (22.037)	
Not	490 (75.734)	115 (69.277)	375 (77.963)	
Intracranial hematoma				0.034
Have	65 (10.046)	23 (13.855)	42 (8.732)	
Not	582 (89.953)	143 (86.145)	439 (91.268)	
Time, median (Q1, Q3)	7.93 (1, 5)	6.16 (1, 4)	8.5 (1, 5)	<0.001
IPR, n%^*^				0.027
Have	37 (5.719)	15 (9.036)	22 (4.574)	
Not	610 (94.281)	151 (90.634)	459 (95.426)	
Therapy method, n%^*^				<0.001
EC	199 (30.757)	41 (24.699)	158 (32.848)	
SAC	381 (58.887)	100 (60.241)	281 (58.420)	
FD	67 (10.355)	25 (15.060)	42 (8.732)	

* AcomA, Anterior communicating artery; PcomA, posterior communicating artery; ACA, anterior cerebral artery; MCA, middle cerebral artery; PCA, posterior cerebral artery; ICA, internal carotid artery; ChoA, choroidal artery; PICA, posterior inferior cerebellar artery; VA, vertebral artery; BA, basilar artery; OA, ophthalmic artery; IPR, intraprocedural rupture; EC, endovascular coiling; SAC, stent-assisted coiling; FD, flow-diverted.

**Figure 1:**
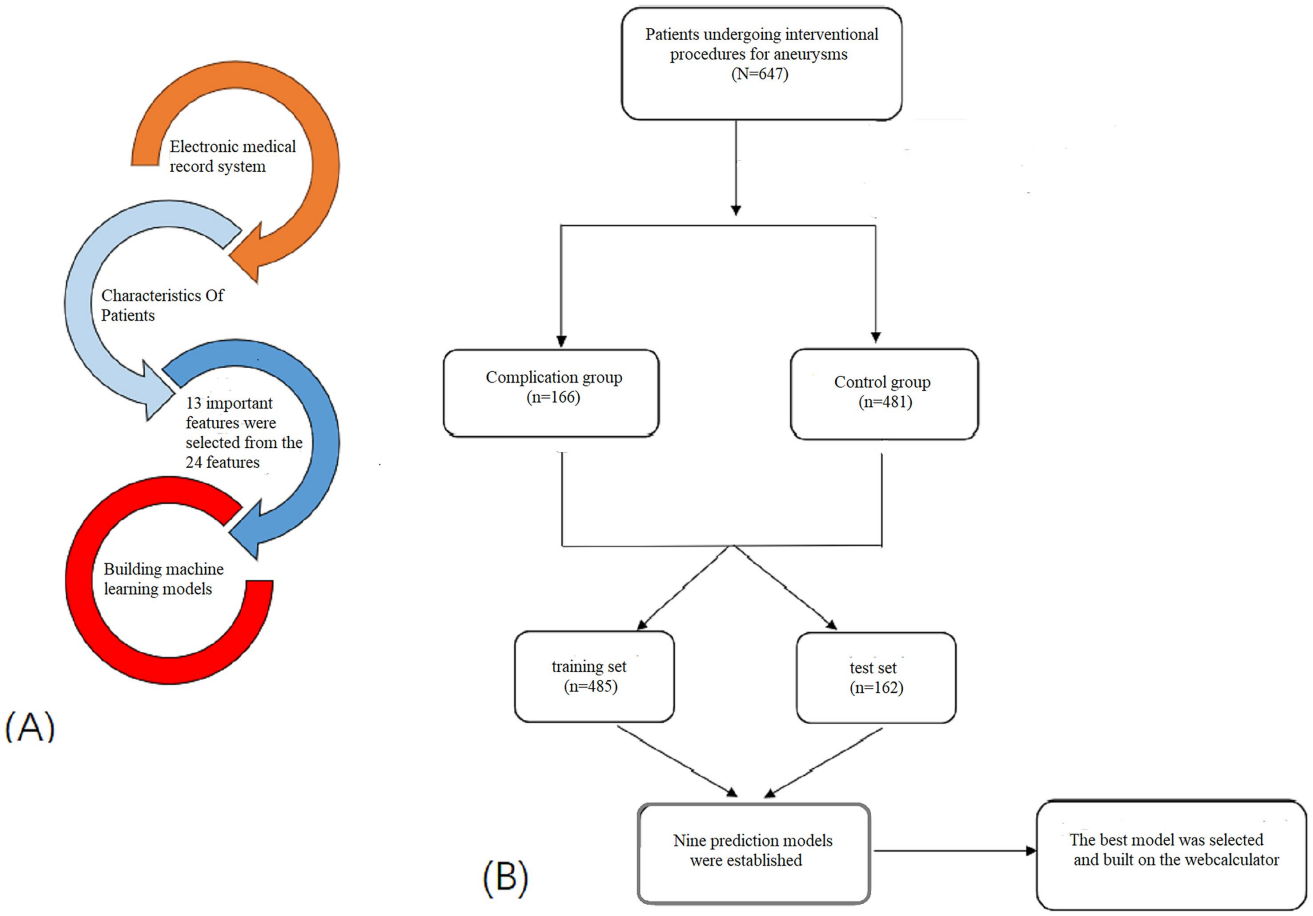
Flow chart of model making process and research procedure. **(A)** The figure shows how to obtain data from the electronic medical record system. A total of 23 variables were collected, and 13 variables were selected to build the machine learning model. **(B)** Study flow chart.

A total of 166 patients (25.66%) experienced PIS within 30 days after endovascular therapy. Following feature screening, all variables exhibited complete data. To develop and validate the prediction model, the study population was randomly divided into a training group (75%, *n* = 485) and a validation set (25%, *n* = 162). Patients were further classified into the complication group (*n* = 166) and the control group (*n* = 481). As depicted in Figure 1, this allocation adhered to a predefined study flowchart. There were no statistically significant differences in patient characteristics between the training and testing datasets.

### 3.2 Important features and model performance

**Figure 2:**
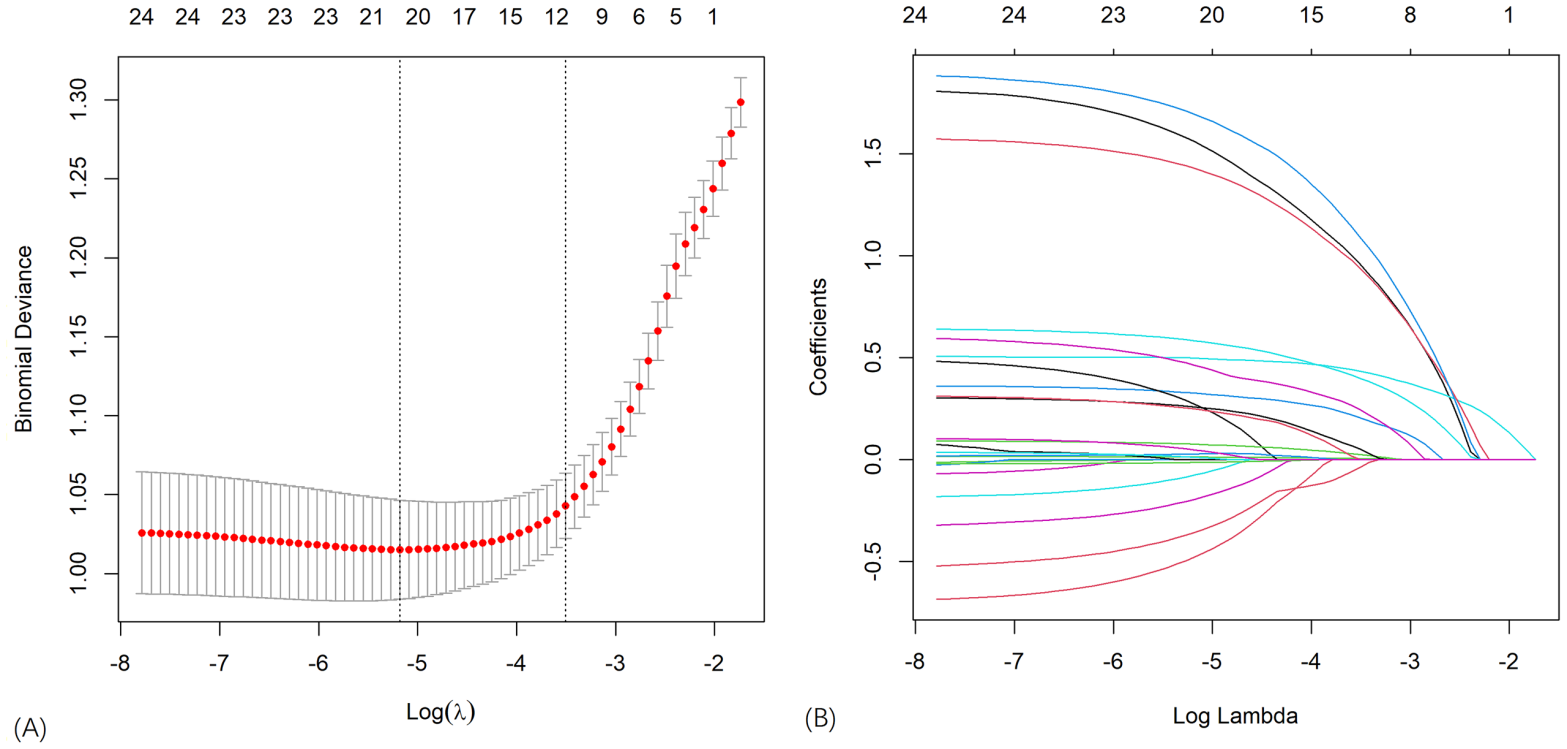
**(A,B)** LASSO regression was used to select the best features.

**Figure 3:**
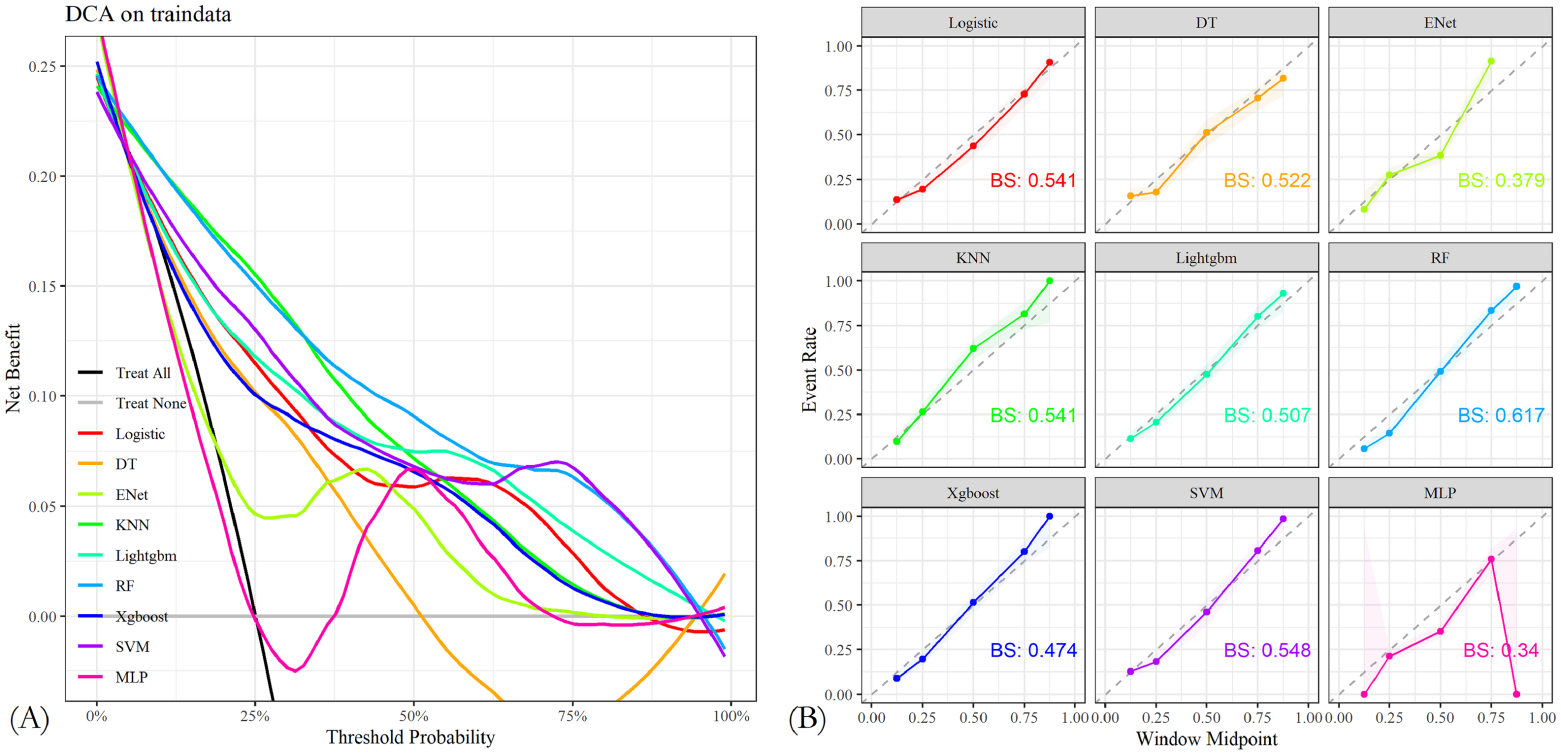
**(A)** Nine machine learning models were analyzed using decision curves. The most effective diagnostic method for perioperative ischemic stroke is the random forest model. **(B)** Calibration plots of the nine models. The random forest model obtained lower Brier scores than the other models.

**Figure 4:**
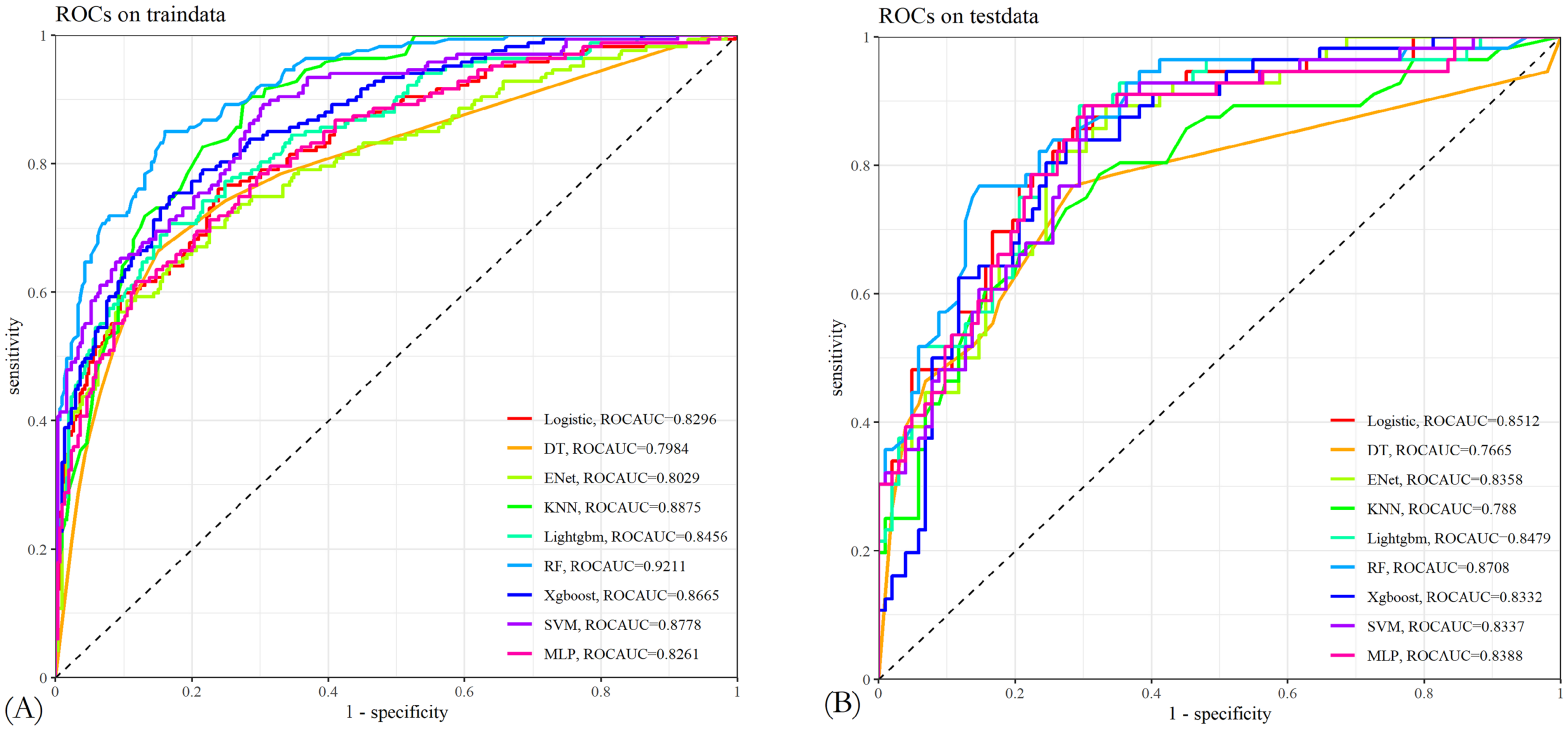
**(A,B)** Characteristic curves of nine machine learning models in the training and test sets. The random forest model had better AUROC.

**Figure 5:**
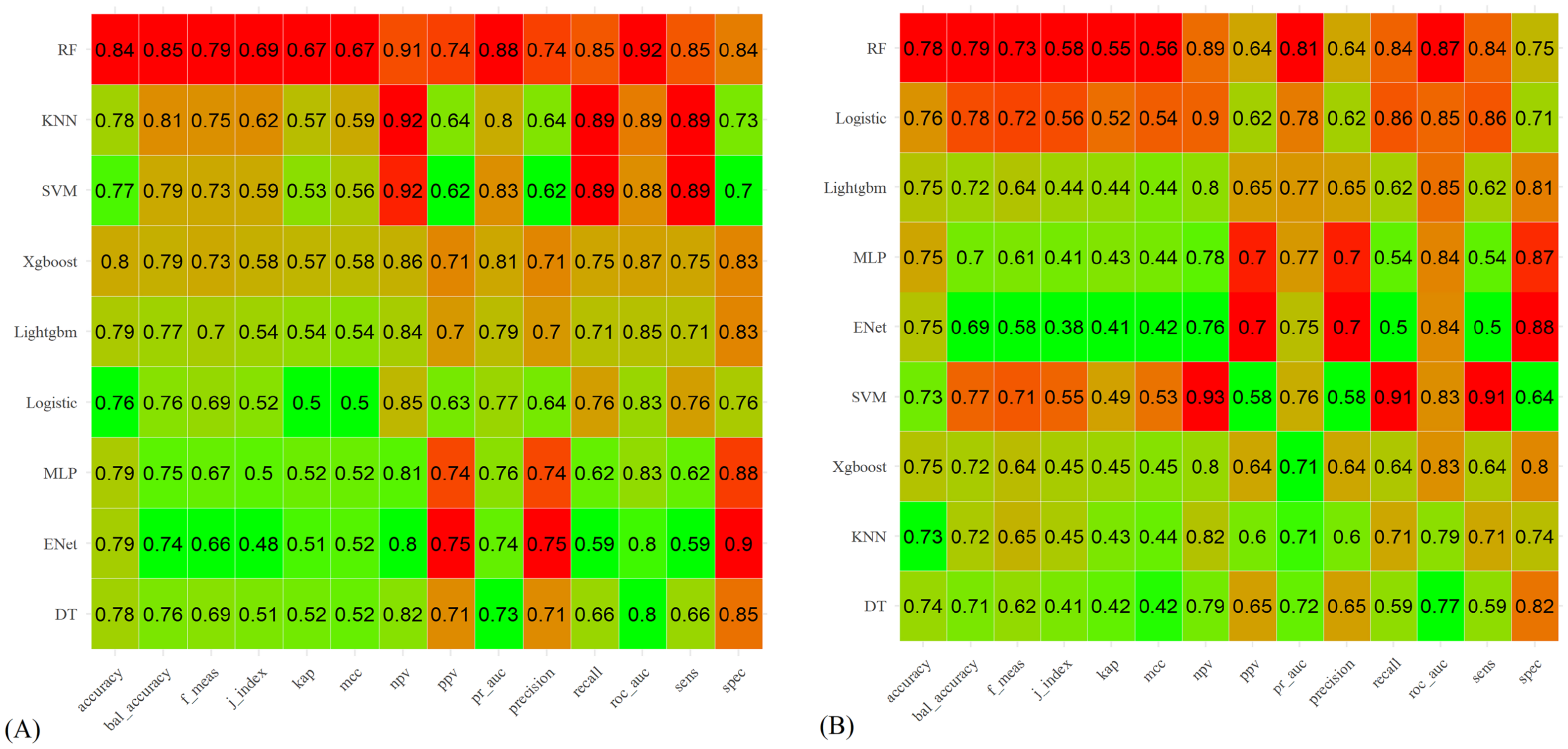
**(A)** Nine machine learning models in the training set and their performance measures. **(B)** Nine machine learning models in the test set and their performance measures.

Through 10-fold cross-validation, the optimal lambda value for LASSO feature selection was determined, resulting in the identification of 10 features with non-zero coefficients (Figures 2A,B). Subsequently, a machine learning model was developed to predict PIS following endovascular procedures using these 13 variables. The performance of the predictive model was assessed using DCA, precision-recall area under the curve (PRAUC), ROCAUC, and Brier score shown in Figures 3, 4. The RF model demonstrated higher ROCAUC and PRAUC values and a lower Brier score compared to other models. Ten cross-validations were performed on all nine models. Figure 5 displays the performance metrics for the training set and test data. The RF model outperformed the other eight models. Additionally, DCA confirmed that RF is the best diagnostic tool for predicting PIS (Figure 3A). The Youden index indicates that a cutoff probability of 36.66% is ideal for the RF. The performance of the established RF model was evaluated using data from the test set (*n* = 162), with the following results: ROCAUC = 87.08% (95% CI: 81.23–92.93%), accuracy = 0.78, balanced accuracy = 0.79, index = 0.58, kap = 0.55, sensitivity = 0.84, specificity = 0.75, and F1 Score = 0.73.

**Figure 6:**
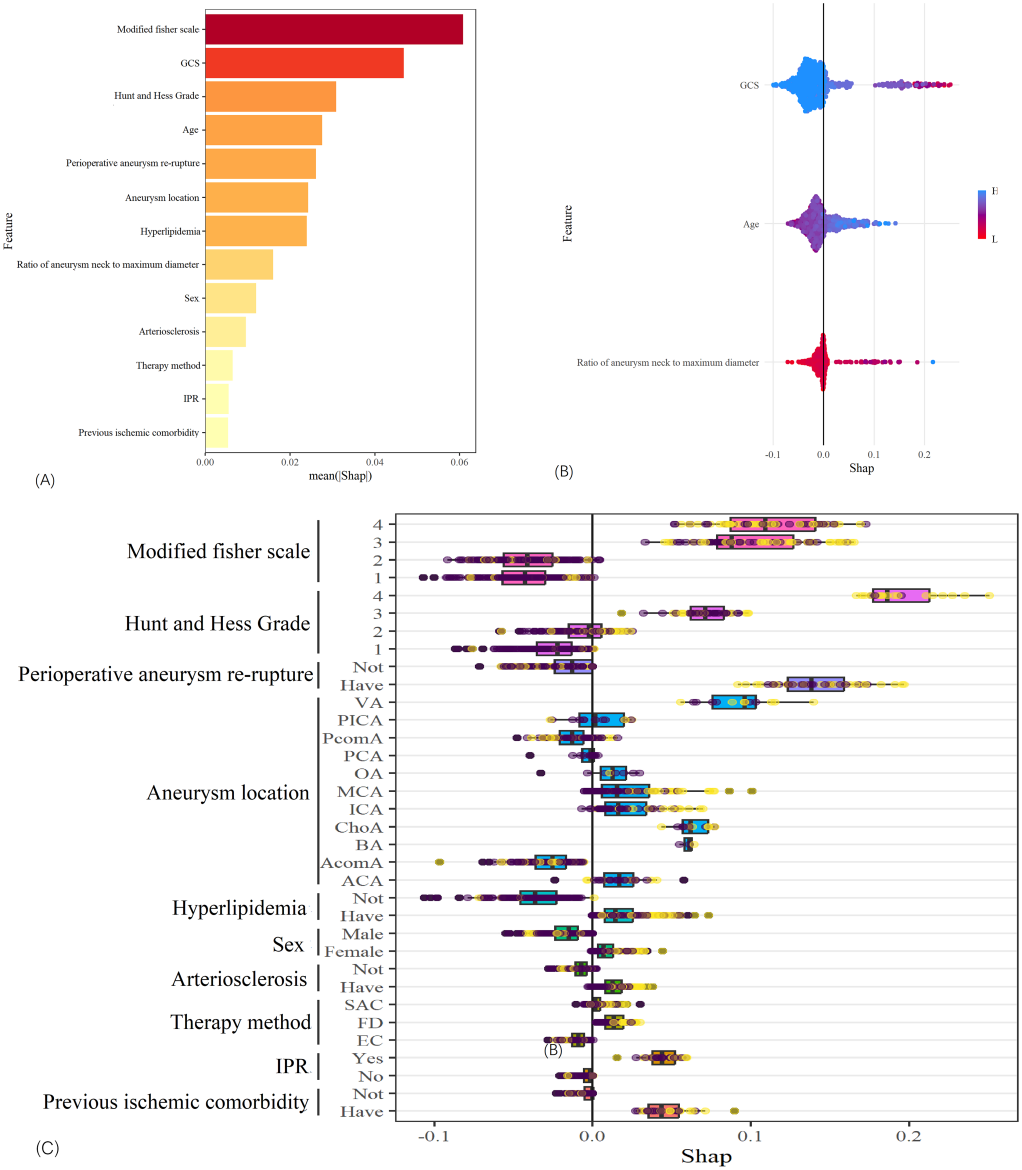
SHAP analysis of the random forest model. **(A)** Shows the relationship between the importance of each feature. **(B)** Shows the relationship between continuous variables and PIS. **(C)** Shows the relationship between classified variable and PIS.

Furthermore, the established RF model was analyzed using the SHAP software package, as depicted in Figure 6. This analysis illustrates the magnitude of the impact of continuous and categorical variables on PIS in the sample using hive plots and box plots, respectively, and the correlation between the size of the eigenvalues and the predicted effects using bar plots.

### 3.3 Model application

**Figure 7:**
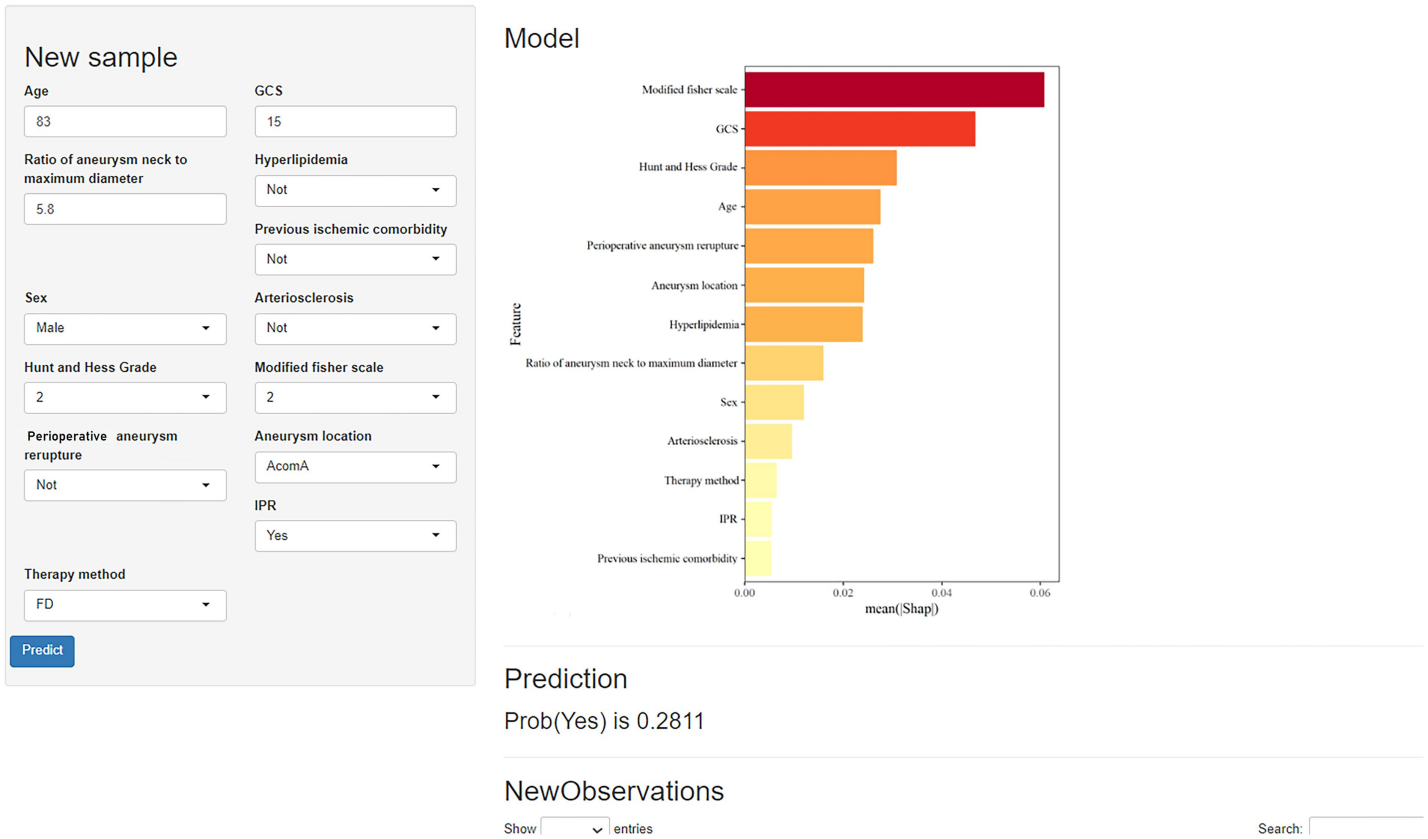
Prediction model of perioperative ischemic stroke in the neuroscience center of the Second Affiliated Hospital of Chongqing Medical University.

Using the shiny package, the developed RF model has been integrated into a web-based application for practical use. Users can input patient data into the model following the instructions provided in the figure. For example, for a male patient aged 83 with an aneurysm located in an AcomA, no arteriosclerosis, no hyperlipidemia, no previous ischemic comorbidity, GCS score of 15, modified Fisher scale of 2, Hunt and Hess Grade of 2, ratio of aneurysm neck to maximum diameter of 5.8, no IPR and treated with FD stents (Figure 7), the model estimated a 28.11% probability of PIS.

It is important to note that the best prediction probability for this model may be truncated to 19.40%. Therefore, patients with a predicted probability exceeding 36.66% are considered at higher risk for PIS. Medical professionals and caregivers should closely monitor individuals with these characteristics and take appropriate actions. Additionally, a web-based calculator based on the proposed model was developed for use by physicians. It is accessible at: Prediction model of perioperative ischemic stroke in the neuroscience center of the Second Affiliated Hospital of Chongqing Medical University (shinyapps.io).

### 3.4 Prospective study to validate

The data of 34 patients were prospectively collected for verification, with 23.53% (8 out of 34) of these patients developing PIS. The model achieved an accuracy of 88.23% on the validation dataset. Notably, all four patients with a predicted PIS probability above the predefined threshold subsequently developed complications, yielding a positive predictive value (PPV) of 100%. Conversely, among patients classified as low-risk, four false-negative cases were identified, while the remaining patients remained complication-free.

## 4 Discussion

The occurrence of PIS following endovascular therapy is a critical concern for practitioners. This study selected the best model as the RF model through the method of machine learning, which has good accuracy (84%) and sensitivity (85%) in identifying PIS. On this basis, a simple and easy-to-operate online calculator was developed, providing clinicians with an efficient and scientific method and tool for comprehensively predicting and evaluating the risk of PIS in RAI patients after endovascular therapy, and offering a preliminary theoretical basis for targeted treatment.

RIAs often lead to subarachnoid hemorrhage (SAH), which can predispose to cerebral vasospasm (CVS), with over 50% of CVS patients being at risk of developing ischemic nerve damage (28), thereby increasing the likelihood of cerebral ischemia. However, quantifying CVS poses challenges. Frontera et al. demonstrated that the Modified Fisher scale was more accurate in predicting symptomatic vasospasm after SAH (29). Therefore, using the Modified Fisher scale as an evaluation of CVS is also a good choice. Meanwhile, studies have shown that the modified Fisher score, Hunt and Hess classification, and GCS are reliable indicators for evaluating the prognosis of patients (30). Therefore, this study investigated their relationship with PIS. As depicted in Figure 6C, the Modified Fisher scale and Hunt and Hess grade showed a strong positive correlation with the occurrence of PIS, whereas the GCS exhibited a strong negative correlation with the occurrence of PIS. In this study, the incidence of PIS was 7.97% for grade I, 23.68% for grade II, 45.00% for grade III, and 61.73% for grade IV. Different Hunt and Hess grades also demonstrated varying incidence rates of PIS: 10.75% for grade I, 30.35% for grade II, 52.38% for grade III, and 72.00% for grade IV, with statistically (*p* < 0.001). According to the latest guidelines (31), a higher modified Fisher scale and Hunt and Hess Grade indicate that earlier surgical intervention may be beneficial in reducing the occurrence of cerebral ischemia and improving functional outcomes. Therefore, successful operation as early as possible may be an effective method to reduce the incidence of PIS in clinical studies.

Perioperative aneurysm re-rupture is one of the most serious adverse events in the endovascular treatment of aneurysms. Existing studies have reported that it can increase the incidence of PIS in patients with RIAs (32). According to various studies, several mechanisms may contribute to perioperative re-rupture. First, aneurysm rupture may be linked to variations in vascular fragility (33). Furthermore, mechanical disruption of the aneurysmal wall may occur during therapeutic embolization procedures due to device-tissue interactions, particularly involving neurointerventional instruments such as microguidewires, microcatheters, or embolization coils. This iatrogenic trauma predisposes to IPR, particularly in cases with fragile vascular architecture (34, 35). The size of the instrument used may be related to the size of the perforation as well as the prognosis. The morbidity and mortality of small puncture wounds caused by micro-guidewires with a diameter of approximately 0.33 mm are lower than those caused by coils or microcatheters (36). Microcatheters with diameters of 0.5–1.0 mm and coils with diameters similar to those of micro-guidewires tend to produce more aneurysm wall tears (35, 36). Coils 2–20 mm in diameter lead to larger perforations and may increase PIS morbidity and mortality. Third, simple intra-aneurysm injection of contrast agent may also induce perioperative aneurysm re-rupture, and the sudden increase in arterial pressure caused by patient pain may also be an important factor in perioperative aneurysm re-rupture (33–35). Therefore, effectively preventing the occurrence of perioperative aneurysm re-rupture may be an effective preventive measure to reduce the occurrence of PIS in patients with RIAs.

Interestingly, this study also found that the location of the aneurysm was one of the most crucial factors in the RF model. The rate of PIS in aneurysms at different locations is also different: AcomA was 20.78%, PcomA was 24.15%, ACA was 31.25%, MCA was 28.43%, PCA was 13.33%, ICA was 23.61%, ChoA was 47.06%, PICA was 20.00%, VA was 57.14%, BA was 44.44%, OA was 12.50%, and the difference was statistically significant (*p* < 0.001). The latest research shows that the incidence of symptomatic cerebral vasospasm after surgery varies among RIA patients with aneurysms at different locations (37). Since cerebral vasospasm often leads to ischemic stroke, the probability of occurrence of PIS is different. Meanwhile, Kocur et al. found that the difficulty of establishing surgical access for aneurysms at different locations varies, thereby resulting in different probabilities of vascular injury and spasm (38). It can be seen from this that endovascular therapy should be carried out by more experienced doctors or more carefully, which may be an effective measure to reduce the occurrence of PIS.

Meanwhile, studies have shown that different endovascular treatment methods (EC, SAC, and FD) for IAs cause different ischemic events, and thus, the probability of developing PIS also varies (39–41). During EC treatment, potential sources of embolization include fragile plaques, iatrogenic dissection in the parent vessel, air bubbles, and materials such as hydrophilic coating materials on catheters and guide wires during insertion or contrast media injection (9, 42, 43). SAC and FD procedures often involve larger-diameter assisted catheters and longer procedure times, which increases the risk of vascular embolism caused by catheters and mechanical operations (44–46). Additionally, the experience of the endovascular neurosurgeon plays a crucial role; less experienced surgeons may require longer procedure times, potentially increasing the risk of intraoperative thrombosis (47). In this study, the incidence of PIS differed among treatment groups: EC (20.60%), SAC (26.25%), and FD (37.31%). Therefore, prior to aneurysm intervention, individualized selection of interventional stent types or whether to use stents for different patients, and minimizing the operation time and steps during the operation, may be an effective strategy to reduce the occurrence of PIS.

In this study, machine learning techniques were used to develop a predictive model for PIS following interventional treatment of RIAs, using nine distinct prediction models. The RF model, identified as the most effective, provides clinicians with enhanced insights into the likelihood of PIS occurrence among different patients. Identifying high-risk patients allows proactive implementation of appropriate preventive measures aimed at reducing PIS incidence and alleviating the associated medical insurance burden (48).

## 5 Limitations

Our study has several limitations that warrant consideration. First, despite our dataset comprising over 600 patients, the data were sourced from a single center, potentially limiting the generalizability of our findings. Furthermore, while we analyzed multiple key factors, there may be additional variables that were not included in our analysis, which could impact the predictive accuracy of our model. Second, the size of our research sample for developing the prediction model was relatively small. Larger datasets are essential for robust training and testing of predictive models to enhance reliability and applicability in clinical settings. Finally, the data used in this study were derived solely from a single center, which may not yield consistent results when applied to different institutions. Future research efforts will focus on collecting extensive datasets and incorporating diverse features from multiple centers to refine the model and optimize its suitability for broader clinical applications.

## 6 Conclusion

PIS stands as the most prevalent complication associated with the endovascular treatment of aneurysms. In our study, we developed nine distinct prediction models for PIS, ultimately selecting the RF model as the optimal predictor based on its superior performance across various metrics in both training and test datasets. The RF model consistently demonstrated the lowest Brier score and high accuracy compared to other classifier models evaluated. By deploying this model and creating a user-friendly web-based calculator, we anticipate it will serve as a valuable tool in clinical practice. This technology enables healthcare providers to effectively identify patients at heightened risk of PIS during endovascular treatment, facilitating the timely implementation of appropriate preventive measures. This approach can enhance the application scope and practicability of risk prediction models, which will enable clinicians to make decisions with more adequate data support, thereby improving patient prognosis and reducing the incidence of PIS.

## Data Availability

The raw data supporting the conclusions of this article will be made available by the authors without undue reservation.
