# Supplementary figures and images for: Automated machine learning for predicting perioperative ischemia stroke in endovascularly treated ruptured intracranial aneurysm patients
Source: Front Neurol. 2025 Jun 19;16:1599856. doi: 10.3389/fneur.2025.1599856 (PMC12222305; doi:10.3389/fneur.2025.1599856)

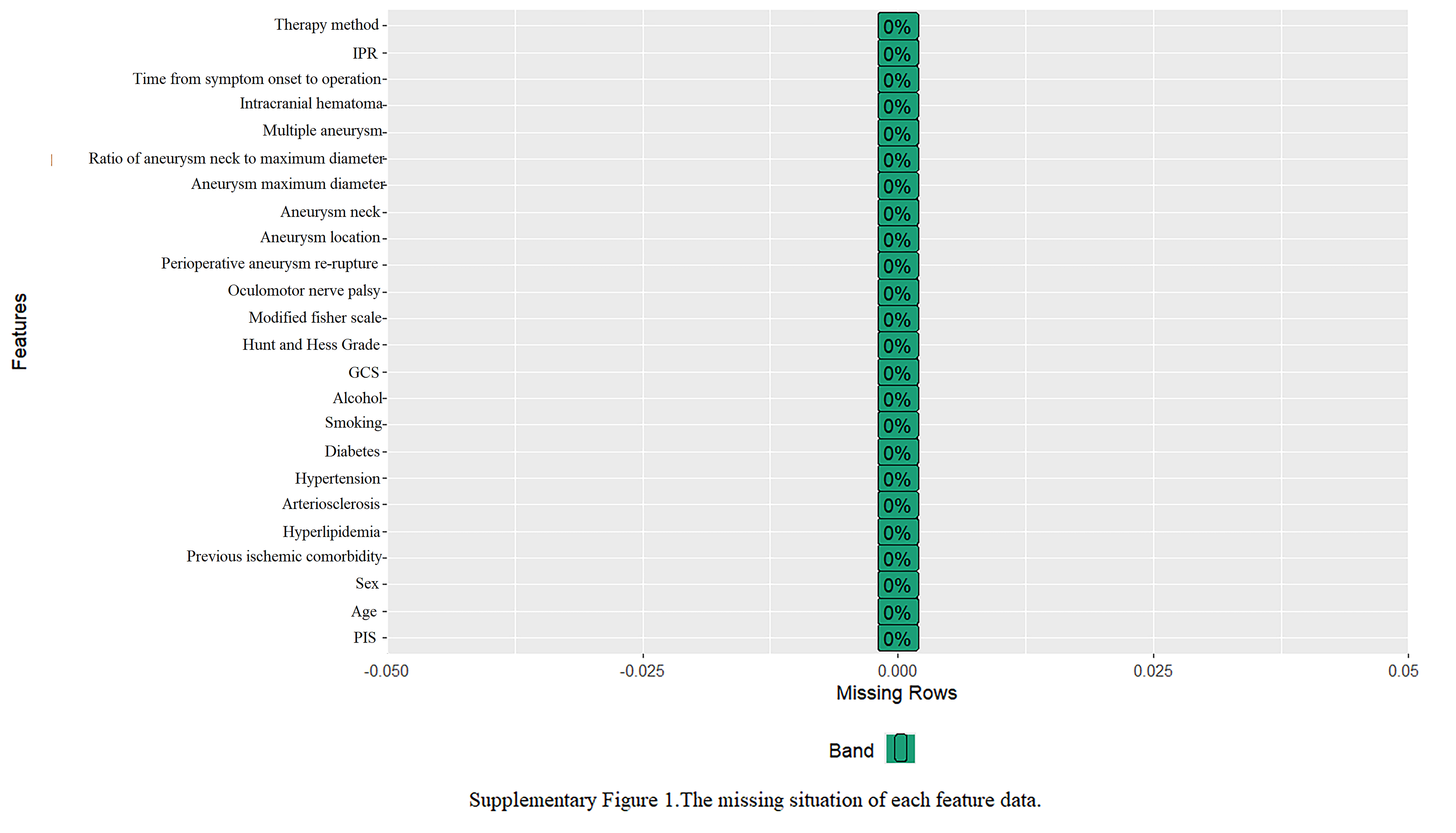

Supplement: Supplementary file 1 [file Image_1.jpeg]
